# Supplementary material for: Categorical consistency of parity and magnitude facilitates implicit learning of color-number associations
Source: PLoS One. 2025 Sep 25;20(9):e0331960. doi: 10.1371/journal.pone.0331960 (PMC12463211; doi:10.1371/journal.pone.0331960)
Supplement: S1 Fig — (PDF) [file pone.0331960.s001.pdf]

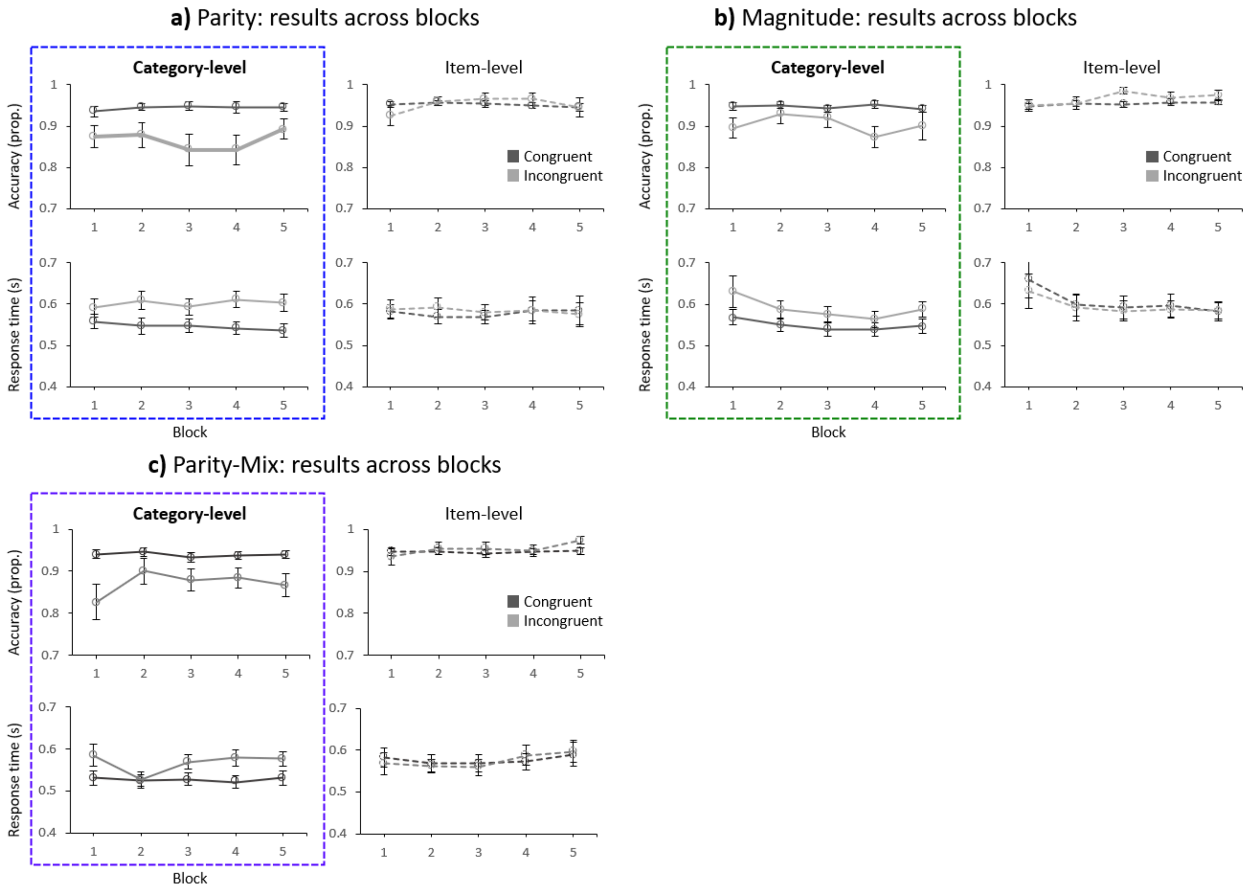

Figure S1. *Performance in terms of accuracy and response time across experimental blocks (5 per experiment part), for Parity (**a**), Magnitude (**b**), and Parity-Mix (**c**).*
