# Supplementary material for: Categorical consistency of parity and magnitude facilitates implicit learning of color-number associations
Source: PLoS One. 2025 Sep 25;20(9):e0331960. doi: 10.1371/journal.pone.0331960 (PMC12463211; doi:10.1371/journal.pone.0331960)
Supplement: S2 Fig — These participants are not outliers, and removing their data has little effect on the pattern of the results. (PDF) [file pone.0331960.s002.pdf]

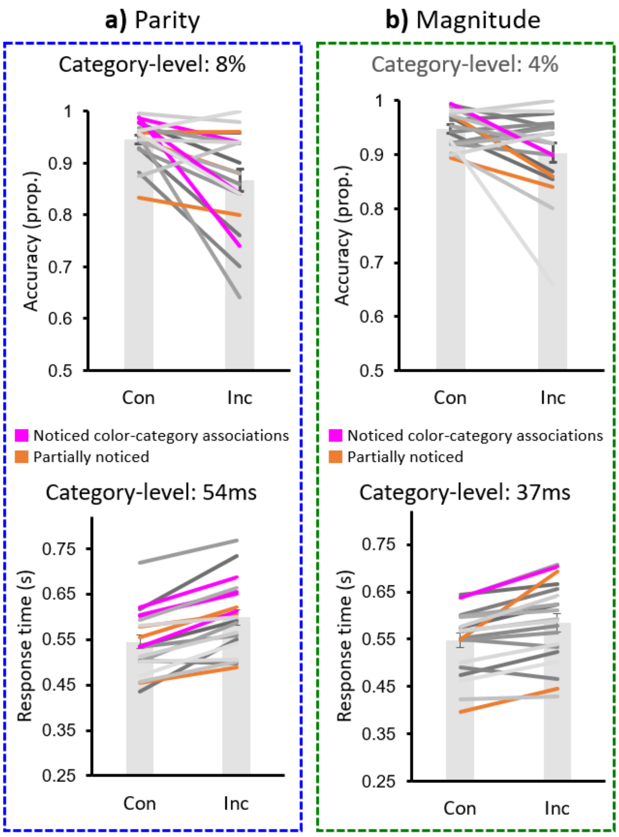

Fig. S2. *Experimental data for participants reporting an awareness of color-category associations: participants correctly reporting the color association for both categories are highlighted in pink; participants partially noticing a color association between color and a category are highlighted in orange. These participants are not outliers, and removing their data has little effect on the pattern of the results.*
