# Supplementary material for: Categorical consistency of parity and magnitude facilitates implicit learning of color-number associations
Source: PLoS One. 2025 Sep 25;20(9):e0331960. doi: 10.1371/journal.pone.0331960 (PMC12463211; doi:10.1371/journal.pone.0331960)
Supplement: Attachment — Submitted filename: PONE-D-25-18078_Review.pdf [file pone.0331960.s003.pdf]

Review of „Categorical consistency of parity and magnitude facilitates implicit learning of color-number associations" – PONE-D-25-18078

General Assessment

This manuscript presents a series of well-designed experiments investigating how number judgments (parity or magnitude) are facilitated when the numbers are consistently colored according to the target number's category (odd/even, small/large number) vs. simply consistent at the item level. The authors replicate and extend previous results, finding clear evidence for superiority of associative learning at the category level vs. the item level. The topic is relevant to cognitive psychology, with implications for theories of incidental associative learning and the interpretation and generalizability of it.

The manuscript is largely well written, provides all necessary methodological details, adequate analyses and addresses a meaningful research question. However, several aspects of the write-up and interpretation/discussion should be improved before warranting publication.

Major Strengths

1. **Convincing Evidence of Category-Level Effects**
   The consistent and statistically robust findings for category-level congruency effects in accuracy and RTs strongly support the idea that this association is superior to mere item-level associations.
2. **Replication and Extension**
   The study replicates prior findings and meaningfully extends them to magnitude associations, which strengthens the reliability and generalizability of the reported effects.
3. **Extensive Post-Experiment Questionnaires**
   The systematic probing of subjects for learned associations with different response keys/hand allows for a thorough picture on the explicitness of the presented associations.

Major Weaknesses and Suggestions for Improvement

1. **Response learning as component in congruity effects**
   The authors mention that response learning (color-response) instead of semantic learning (color-number) could explain the congruity effects. However, when interpreting the results, the authors nevertheless seem to stress the semantic interpretation, based on the fact that „Participants were able to explicitly report the category-level, and item-level parity, color associations above chance-level, with different response keys/hands, suggesting that this associative learning occurred beyond the response-level" (p. 26).
   While I concur with this interpretation (there is something happening beyond the response level), I think it is warranted to discuss in more depth that the results in the main experimental tasks itself could still simply be explained by participants learning to associate a certain color with a response key (J vs. K), a response finger (index vs. middle), or a response side (left vs. right) etc. In fact, the category-level task can easily be performed by simply responding to yellow numbers with the J button, blue numbers with the K button

(except incongruent trials; similarly for magnitude). The main experiment is simply not designed to distinguish between semantic and response learning. Previous studies have however explicitly distinguished these two options, for example Schmidt and Cheesman (2005), or Geukes, Vorberg, and Zwitserlood (2019). So, it would helpful to discuss this possibility more thoroughly and to suggest ways in which this could be distinguished in future studies.
Interestingly, the strategy of relying on the color to pick the response naturally fails in the item-level task, which could straightforwardly explain the missing effects there. Hence, I think this distinction should be discussed in more depth: what can and what cannot be concluded from the main experiment in terms of response vs. semantic learning? And what can the (very helpful) post-experiment tests add to this?

2. **Missing Counterbalancing**
The authors cleanly separated colors and response hands between the parity and magnitude main experiments to avoid carry-over effects. But, as far as I could gather, throughout the experiments, these and several other factors could still have been – but were not – counterbalanced between participants: assignment of response buttons to response categories (e.g., J is always odd, K is always even for all participants), order of experiments (magnitude always follows parity), color to parity/magnitude assignment (e.g., even numbers are always blue, smaller numbers are always green for all participants). All these fix assignments can introduce unwanted associative effects that could easily be reduced by counterbalancing. What was the rationale to not counterbalance these factors? The rationale should be mentioned, and the potential impact discussed in more detail (the authors only briefly allude to a potential impact of the fix color assignment, on page 21).

3. **Statistical Reporting**
The authors provide a comprehensive and well-structured results section. Nevertheless, I think this section is quite hard to read and should be simplified a little. For example, overall congruity effects ignoring the category- and item-levels are not that interesting to report and could be omitted, e.g. this sentence: „The main effect of Congruency was also significant for both accuracy, $F_{1,38} = 11.8$, $p = .001$, eta_p squared = 0.24, and RT, $F_{1,38} = 36.1$, $p < .001$, eta_p squared= 0.49." (In the same way as the authors do not report the main effect of LEVEL).
The mentioned tests for the difference in the size of the congruency effects between experiment levels indeed MUST be omitted because they are mathematically redundant (e.g., the sentence „The difference in congruency effect was significant across experiment levels for both accuracy, $t_{24.7} = 3.51$, $p < .001$, $d = 1.11$, and RT, $t_{38} = 5.05$, $p < .001$, $d = 1.60$." on pdf page 16). These tests are mathematically identical to testing the interaction effect that the authors already report before (e.g., end of page 15): „There was a significant interaction between Congruency and Level, for both accuracy, $F_{1,38} = 12.3$, $p = .001$, eta_p squared= 0.25, and RT, $F_{1,38} = 25.5$, $p < .001$, eta_p squared = 0.40.".
The t value squared from this test of differences is identical to the F-value from the interaction test: 3.51 squared = 12.3, therefore the test provides the exact same inference test. In fact, I could confirm that for both Parity and Parity-Mix, the t squared equal the F. However, I found a deviation for magnitude. So there must be an error with the magnitude reporting. Please double check these results.

4. **Clarity of Discussion**
   The discussion is comprehensive, which I appreciate. But especially the beginning is redundant compared to the results section: the discussion seems to almost repeat the results section with a detailed listing of descriptive and inferential statistics. I think this is not necessary and could be shortened, instead beginning by restating the purpose of the study and then directly interpreting the key results relevant to the research question(s).

Recommendation

Minor Revision
The manuscript offers a valuable contribution to the field and is on track to be suited for publication in PLOS ONE, but it should be revised to sharpen the results presentation and the interpretation and discussion of the findings.

Minor Comments
- pdf page 12, line 6: typo „dived" should be „divided“
- which version of Cohen’s d are you reporting? Cohen’s d is a measure for a between samples t-test. For within-samples t-tests, there are different definitions for a variant of Cohen’s d (Goulet-Pelletier & Cousineau, 2018). Please mention which one you are reporting.

References
Geukes, S., Vorberg, D., & Zwitserlood, P. (2019). Disentangling semantic and response learning effects in color-word contingency learning. *PLOS ONE, 14*(5), e0212714. https://doi.org/10.1371/journal.pone.0212714

Goulet-Pelletier, J.-C., & Cousineau, D. (2018). A review of effect sizes and their confidence intervals, Part I: The Cohen’s d family. *The Quantitative Methods for Psychology, 14*(4), 242–265. https://doi.org/10.20982/tqmp.14.4.p242

Schmidt, J. R., & Cheesman, J. (2005). Dissociating Stimulus-Stimulus and Response-Response Effects in the Stroop Task. *Canadian Journal of Experimental Psychology/Revue canadienne de psychologie expérimentale, 59*(2), 132–138. https://doi.org/10.1037/h0087468
