# Supplementary material for: Categorical consistency of parity and magnitude facilitates implicit learning of color-number associations
Source: PLoS One. 2025 Sep 25;20(9):e0331960. doi: 10.1371/journal.pone.0331960 (PMC12463211; doi:10.1371/journal.pone.0331960)
Supplement: Attachment — Submitted filename: ParityMagnitude_PLOSONE_reviewd_CS_final.docx [file pone.0331960.s004.docx]

**Reviewers' comments:**

**Reviewer #1:**

“Categorical consistency of parity and magnitude facilitates implicit learning of color-number associations” by Retter and Schiltz:
This manuscript provides valuable insights into the relationship between color and parity and color and magnitude, in terms of whether associative learning can result in implicit associations between these dimensions. The authors conducted a single experiment, with three tasks. The parity task aimed to replicate prior findings by the researchers demonstrating associative learning can emerge between color and parity. The magnitude task was a novel task designed to evaluate whether associative learning could also extend to mapping colors to categories of magnitude. And lastly, the parity-mix task aimed to demonstrate whether learning from tasks 1 and 2 could be merged to influence responses in a final parity task. At the end participants were also asked to complete tasks aiming to explicitly demonstrate learned associations.
The data are appropriately analyzed and the majority of the results are clear and carefully describe key details. There are no concerns about potential competing interests on the part of the authors, data availability, or research ethics. Together, this manuscript reports an interesting experimental design investigating parity/magnitude associations. However, there are several points of concern that I think should be addressed to ensure this work is ready for publication, including clarifying the purpose/context of the work amidst prior work and addressing concerns in methodology of part 3.

**Reply: We thank Reviewer 1 for the clear summary and positive evaluation of our manuscript. Please find our responses to the comments below, with changes highlighted in yellow in the accordingly revised manuscript.**

Major concerns
1. The theoretical basis/motivation for this work was limited throughout the introduction. There was a clear overview of associative learning and why investigating associative learning and categorical-level (vs. item-level) is important, which was great. However, there was little discussion on why parity and magnitude are beneficial categories to assess in the context of associative learning.

Similarly, it was unclear throughout the introduction what the rationale was for replicating the authors’ previous work on parity, as well as, why it is important to extend these ideas to magnitude. Specifically, I think the work would benefit from additional elaboration and clarification how this this work is different from the previous studies performed by these authors (i.e., adding to the statement on page 2 of the Intro: “Here, we aimed to replicate these results for parity, and extend the paradigm to testing for another category-level association, magnitude, which is a fundamental aspect of number processing.”).

**Reply: We fully agree with Reviewer 1 that the motivation for studying associative learning with magnitude and parity should be further elaborated in the introduction. We have substantially modified this section, adding a paragraph as to the interest in studying parity (p. 2, from line 38), and better introducing magnitude in comparison (p. 2, from line 48).**

2. The rationale, design, and interpretation of the results of the parity-mix task left me with a number of questions/concerns. The authors mention that all participants completed the tasks in the same order (parity, magnitude, parity-mix), which seems particularly concerning in evaluating the results of this final task. It seems quite possible that order-effects are contributing to the results, such that a primacy effect could be driving why participants were most likely to report blue-yellow colors correctly give those are the color categories first learned in the parity task. This concern could be addressed with additional clarification on the logic for why the order was not balanced across participants and/or, ideally, a set of data in which the order is reversed for tasks 1 & 2.

**Reply: Participants did perform the experiments in a fixed order: the rationale for this is expanded in a new paragraph of the revised manuscript (p. 7). Essentially, this was done so that the Parity-Mix experiment could be presented third (after participants had potentially learned both parity- and magnitude-color associations). The Parity (blue/yellow) experiment was then presented first, so that it was not adjacent to the Parity-Mix (purplish-blue/greenish-yellow) experiment, to keep participants’ perception of the shifted Parity-Mix colors from being affected by immediate exposure to the Parity colors. Instead, these two parity experiments were separated by the Magnitude (purple/green) experiment.**

**In a previous experiment on parity and magnitude, we did counterbalance the order (Retter & Schiltz, 2025, *Journal of Cognition*), but that was not possible here, in consideration of the additional Parity-Mix experiment. We further discuss the limitations of this fixed order in the discussion, in the context of comparing effects across parity and magnitude categories (p. 15), as well as the explicit report task results (p. 19).**

Further, the colors for the parity-mix task read as an essential component to the task. However, given the limited recognition by the participants that the colors were different, this suggests that the task likely appear the exact same as task 1 for participants. If the colors were more distinct, such that the purplish-blue and yellowish-green were clear border colors between purple and blue and yellow and green (and that participants reported such colors as being on their own color category borders), the results would more directly address the question of whether associations are overlapping across categories.

Further, I wonder whether other kinds of associations could be driving the match vs. mismatched results of this task, such as the dark-is-more bias (e.g., McGranaghan (1989), Schloss, et al. 2018; etc.), in which people infer darker colors represent more. Blues and purples are generally/often darker than yellows and greens and were mapped to larger magnitudes in the present study. Such a bias being activated could provide an alternative explanation to the higher accuracy of matched trials than mismatched. The work would benefit from a discussion considering these points in the context of interpreting the results of the parity-mix task.

**Reply: We appreciate Reviewer 1’s attention given to the colors used in the experiment. We thought it was important to use shifted colors that still appeared to be within the categories of blue and yellow (with a parity task in this Parity-Mix experiment), since we wanted to preserve the potential blue/yellow associations formed with parity in the first experiment. If we used boarder blue-purple or green-yellow stimuli, this might have imposed new parity-color associations during the course of this third experiment. We now explain the rationale for this parity “color shift” in the revised manuscript (p. 4). We were surprised that only one participant commented on the different colors in the Parity-Mix experiment (and that the color recall report was poor in general): however, we do not think this means that the Parity-Mix colors appeared the same as in the Parity experiment (discussed further on p. 20).**

**We take the evidence for stronger associative learning for match vs. mismatch numbers as supporting that a (purplish- and greenish-) difference was perceived. In our paradigm, there are two purplish-blue and two greenish-yellow colors in the both the match and mismatch groups, so that we do not think potential prior color associations, such as the dark-is-more bias, could explain these results. However, we take the point that such a bias might affect strength of category-level associations in the magnitude experiment (although, green is actually darker than purple in our stimuli, and we are not actually aware of darkness biases across these categories), and have mentioned this in the revised manuscript (p. 15).**

3. Throughout the introduction (and a bit beyond too), the use of “category-level” could be interpreted with respect to multiple different dimensions, including parity, magnitude, and color. To clarify, when using “categorical-level”, for the most part (as I understand it), the authors’ use it in reference to the categories of parity and/or magnitude and NOT the color categories used (i.e., the color category of blue). However, in the Introduction’s discussion of the parity-mix experiment, there are references to associations extending to different color categories and in the design of that experiment itself, there is greater consideration color categories (given the colors are shifted towards neighboring color categories) as well as the parity and magnitude categories. The manuscript would benefit from ensuring clarity throughout on which categories are being referenced when discussing the category-level. I encourage the authors to provide additional details and examples throughout the introduction to clarify the multiple dimensions being discussed.

**Reply: We apologize that the term “category-level” was not clear in the manuscript: we use this to describe the experiment version in which associations may occur *between* numerical (parity or magnitude) and color categories, e.g., between even-blue and odd-yellow. In contrast, the term “item-level” describes the experiment version in which the two colors categories are not consistent with numerical categories (e.g., 2,3,6,7-blue and 4,5,8,9-yellow). We have stated this explicitly in the revised manuscript (p. 1, line 29). We have also included more mentions of both numerical categories and color categories (e.g., p. 2, lines 40-41).**

4. In order for others to have the ability to replicate this work, it would be necessary to add information on the following:
a. how much time participants took (on average) to complete each task,
b. how much compensation was given out to participants,
c. how participants were evaluated for atypical color-vision (It seems quite surprising not a single participant of 40 had atypical color vision given prevalence rates)
d. the rationale for the sample size of 40 participants (e.g., was a power analysis performed? If not, how was this number of 20 per condition selected and how is sufficient power ensured?)

**Reply: We have added the requested information to the text as follows: a) each implicit associative learning experiment lasted about 15 minutes (p. 5, line 142); the total testing session was 1.5 hours: p. 3; b) participants were paid 15 euros: p. 3; c) participants were only asked about color perception, they were not tested for visual deficits, but normal (or corrected-to-normal) visual acuity and color perception were recruitment inclusion criteria: pp. 3-4; d) the sample size was determined relative to previous implicit association learning studies, particularly the very similar design of Retter, Eraßmy & Schiltz, 2023, PLoS ONE (16 participants per group): p. 3.**

5. In the discussion, the authors briefly discuss one practical extension of the work. Aligning with the comments on the introduction and providing additional motivation for why this work is important, it would be beneficial to expand on the implications from these results.

**Reply: The discussion section has been expanded, including adding a new beginning section orienting the main experimental aims (p. 13), and a new section on “Category-level associative learning for parity vs. magnitude” (p. 14). We have more carefully addressed some limitations of our design (e.g., not isolating potential response-learning: p. 17), as well as newly emphasized the implications (e.g., last sentence of the summary, p. 21).**

Minor concerns
6. Regarding the methods, it is currently unclear how many total congruent versus incongruent trials were presented for each task. This information seems particularly important to include given the results are dependent on the differences between congruent & incongruent.

**Reply: We have explicitly added the number of congruent and incongruent trials to the methods (p. 5, line 137).**

A related minor comment, when reporting the results, would help the readers to see the means & variation for each level being used to determine the congruency effect when discussing this effect (versus currently the means/SE are discussed later in the results). This would be especially helpful given that the individual subject data on the results graphs make it a bit challenging to clearly see the means & error bars.

**Reply: As suggested, we have moved the M and SE reports to the beginning of each results section, when reporting the congruency effect (this change in position is indicated with a yellow highlight only on the beginning of the report, “(congruent: ”, to make it clear that the M and SE data did not change.) pp. 9-11.**

7. While the Supplementary materials are available, a more detailed summary of the main points from those could be incorporated into the main text. Currently, the sentence regarding the “congruency effects were observed to evolve across blocks for the category level experiment….” felt out of place and missing the context needed to understand its importance.

**Reply: We have added a description of the results in Fig. S1 (p. 10). These results are also now referenced for the Parity-Mix experiment (p. 11; p. 19).**

8. There is a bit of inconsistency in the language used throughout the manuscript, that sometimes makes it a bit difficult to interpret the information. For instance, each of the following were used in reference (to at least I think) the same manipulation in the experiment: category-level experiment, category-level version,; experimental group.

**Reply: We agree that our language was inconsistent, and have removed all references of “category-level experiment” throughout. It remains only that participants in the experimental group performed the category-level version of each experiment (e.g., p. 1; p. 6).**

9. The discussion had a few typos (the rest of the paper was well-written in that regard): a few examples include; “to” is missing between “compared” and “incongruent” in the first sentence; “leaning” instead of “learning” , “we predicting” instead of “we predicted,” etc.

**Reply: These typos have been corrected, and we have carefully proofread the discussion. Thank you for this attention to detail.**

**We again thank Reviewer 1 for the constructive comments, with which we were able to considerably improve our manuscript.**

**Reviewer #2:**

General Assessment

This manuscript presents a series of well-designed experiments investigating how number judgments (parity or magnitude) are facilitated when the numbers are consistently colored according to the target number’s category (odd/even, small/large number) vs. simply consistent at the item level. The authors replicate and extend previous results, finding clear evidence for superiority of associative learning at the category level vs. the item level. The topic is relevant to cognitive psychology, with implications for theories of incidental associative learning and the interpretation and generalizability of it. The manuscript is largely well written, provides all necessary methodological details, adequate analyses and addresses a meaningful research question. However, several aspects of the write-up and interpretation/discussion should be improved before warranting publication.

Major Strengths

1. Convincing Evidence of Category-Level Effects

The consistent and statistically robust findings for category-level congruency effects in accuracy and RTs strongly support the idea that this association is superior to mere item-level associations.

2. Replication and Extension

The study replicates prior findings and meaningfully extends them to magnitude

associations, which strengthens the reliability and generalizability of the reported effects.

3. Extensive Post-Experiment Questionnaires

The systematic probing of subjects for learned associations with different response keys/hand allows for a thorough picture on the explicitness of the presented associations.

**Reply: We thank Reviewer 2 for the positive evaluation, including highlighting these strengths of our manuscript.**

Major Weaknesses and Suggestions for Improvement

1. Response learning as component in congruity effects

The authors mention that response learning (color-response) instead of semantic learning (color-number) could explain the congruity effects. However, when interpreting the results, the authors nevertheless seem to stress the semantic interpretation, based on the fact that “Participants were able to explicitly report the category-level, and item-level parity, color associations above chance-level, with different response keys/hands, suggesting that this associative learning occurred beyond the response-level“ (p. 26).

While I concur with this interpretation (there is something happening beyond the response level), I think it is warranted to discuss in more depth that the results in the main experimental tasks itself could still simply be explained by participants learning to associate a certain color with a response key (J vs. K), a response finger (index vs. middle), or a response side (left vs. right) etc. In fact, the category-level task can easily be performed by simply responding to yellow numbers with the J button, blue numbers with the K button (except incongruent trials; similarly for magnitude). The main experiment is simply not designed to distinguish between semantic and response learning. Previous studies have however explicitly distinguished these two options, for example Schmidt and Cheesman (2005), or Geukes, Vorberg, and Zwitserlood (2019). So, it would helpful to discuss this possibility more thoroughly and to suggest ways in which this could be distinguished in future studies.

Interestingly, the strategy of relying on the color to pick the response naturally fails in the item-level task, which could straightforwardly explain the missing effects there. Hence, I think this distinction should be discussed in more depth: what can and what cannot be concluded from the main experiment in terms of response vs. semantic learning? And what can the (very helpful) post-experiment tests add to this?

**Reply: We think this is a good point, and have added a discussion of potential response-level learning effects in the revised manuscript; we now mention that our experiments were not designed to isolate potential response-level learning effects, unlike those of previous studies (Schmidt & Cheesman, 2005; Geukes, Vorber & Zwisterlood, 2019) (p. 17).**

**We also acknowledge that in our design color correlated with the response keys with a high-probability in the category-level experiment. However, we do not think that there is evidence that in addition to evaluating the parity or magnitude of the number, participants also evaluated its color: to the contrary, only four participants correctly reported an awareness of the color association of the numbers with a numerical category. The simple numerical tasks were done rapidly, with high accuracy, similarly by participants in both the category-level and item-level versions of the experiments (Fig. 2). This is also now addressed in the revised manuscript (p. 17).**

2. Missing Counterbalancing

The authors cleanly separated colors and response hands between the parity and

magnitude main experiments to avoid carry-over effects. But, as far as I could gather, throughout the experiments, these and several other factors could still have been – but were not – counterbalanced between participants: assignment of response buttons to response categories (e.g., J is always odd, K is always even for all participants), order of experiments (magnitude always follows parity), color to parity/magnitude assignment (e.g., even numbers are always blue, smaller numbers are always green for all participants). All these fix assignments can introduce unwanted associative effects that could easily be reduced by counterbalancing. What was the rationale to not counterbalance these factors? The rationale

should be mentioned, and the potential impact discussed in more detail (the authors only briefly allude to a potential impact of the fix color assignment, on page 21).

**Reply: Considering the order of experiments, we have explained the fixed order carefully in the revised methods (p. 7): essentially, this is to finish the implicit associative learning experiments with the parity/magnitude combined Parity-Mix experiment. We address potential impacts of not having counter-balancing the order, and colors, in the revised discussion (p. 15). Response keys were not counter-balanced for simplicity, given that no response key biases were predicted a priori, and to exactly replicate the previous study (p. 5, line 150).**

3. Statistical Reporting

The authors provide a comprehensive and well-structured results section. Nevertheless, I think this section is quite hard to read and should be simplified a little. For example, overall congruity effects ignoring the category- and item-levels are not that interesting to report and could be omitted, e.g. this sentence: “The main effect of Congruency was also significant for both accuracy, F1,38 = 11.8, p = .001, eta_p squared = 0.24, and RT, F1,38 = 36.1, p < .001, eta_p squared= 0.49.“ (In the same way as the authors do not report the main effect of LEVEL).

**Reply: We have accordingly removed reporting the main effects when there was a significant interaction. In response to Reviewer 1 (comment 6), we have also moved a description of the accuracy and RT values earlier in the results, when reporting the congruency effects, to further improve the readability.**

The mentioned tests for the difference in the size of the congruency effects between

experiment levels indeed MUST be omitted because they are mathematically redundant (e.g., the sentence „The difference in congruency effect was significant across experiment levels for both accuracy, t24.7 = 3.51, p < .001, d = 1.11, and RT, t38 = 5.05, p < .001, d = 1.60.“ on pdf page 16). These tests are mathematically identical to testing the interaction effect that the authors already report before (e.g., end of page 15): „There was a significant interaction between Congruency and Level, for both accuracy, F1,38 = 12.3, p = .001, eta_p squared= 0.25, and RT, F1,38 = 25.5, p < .001, eta_p squared = 0.40.“. The t value squared from this test of differences is identical to the F-value from the interaction test: 3.51 squared = 12.3, therefore the test provides the exact same inference test. In fact, I could confirm that for both Parity and Parity-Mix, the t squared equal the F. However, I found a deviation for magnitude. So there must be an error with the magnitude reporting. Please double check these results.

**Reply: We removed the t-tests on the congruency effects across experiment levels. We apologize for that redundancy, and agree that this analysis indeed should have been removed. We also apologize for the error in the magnitude reporting, which mistakenly contained some values from the Parity-Mix experiment: thank you for bringing this to our attention (corrected significant magnitude accuracy interaction: p. 10, line 283). These actual statistics indeed match the reported t-test (F = 10.63 = 3.26 squared); the t-value of the magnitude RT congruency effect (test removed from the revised manuscript) was actually 4.09, not 4.45 (4.09 squared equals the F value of 16.73.) We have double checked all reported statistics and result values to ensure accurate reporting, thank you for these careful observations which have led to these important corrections.**

4. Clarity of Discussion

The discussion is comprehensive, which I appreciate. But especially the beginning is

redundant compared to the results section: the discussion seems to almost repeat the results section with a detailed listing of descriptive and inferential statistics. I think this is not necessary and could be shortened, instead beginning by restating the purpose of the study and then directly interpreting the key results relevant to the research question(s).

**Reply: We agree that the discussion could use some improvement, and was too repetitive with the results section. The discussion has been substantially revised, including introducing a new paragraph at the beginning, outlining the three main aims of the study that will be addressed (p. 13). These research questions are also better answered in concluding sentences of each section (e.g., p. 14). Although the section has not been shortened, we hope that this reorganization has made it more focused, we have also removed restatements of the statistical results throughout.**

Recommendation

Minor Revision

The manuscript offers a valuable contribution to the field and is on track to be suited for publication in PLOS ONE, but it should be revised to sharpen the results presentation and the interpretation and discussion of the findings.

Minor Comments

• pdf page 12, line 6: typo „dived" should be „divided“

**Reply: This typo has been corrected.**

• which version of Cohen’s d are you reporting? Cohen’s d is a measure for a between samples t-test. For within-samples t-tests, there are different definitions for a variant of Cohen’s d (Goulet-Pelletier & Cousineau, 2018). Please mention which one you are reporting.

**Reply: The Cohen’s d for paired samples t-tests is as reported in SPSS 28, using the sample standard deviation of the mean difference; this calculation indeed differs for within and between samples tests (details are reported in the SPSS 28 manual, p. 1240:** [**https://www.ibm.com/docs/en/SSLVMB_28.0.0/pdf/IBM_SPSS_Statistics_Algorithms.pdf**](https://www.ibm.com/docs/en/SSLVMB_28.0.0/pdf/IBM_SPSS_Statistics_Algorithms.pdf)**).**

**We thank Reviewer 2 again for the helpful comments, which in following we were able to importantly correct and improve our manuscript.**
